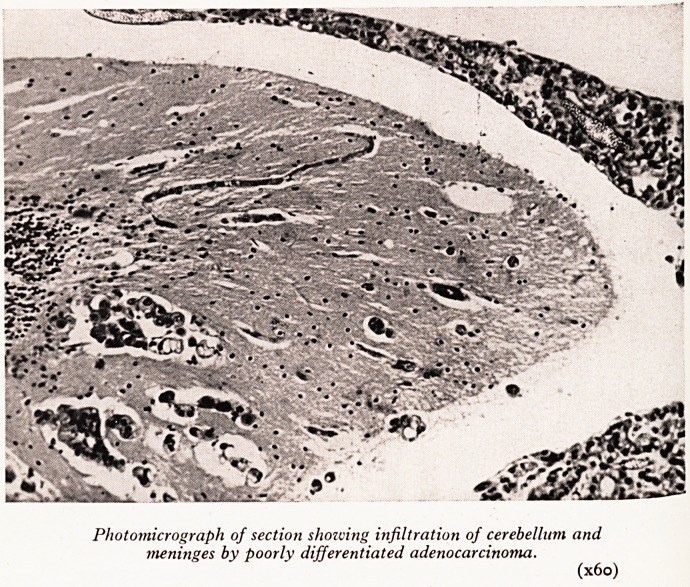# A Case of Meningeal Carcinomatis

**Published:** 1960-10

**Authors:** O. C. Lloyd


PLATE XV

*Smear from C.S.F. deposit, showing collection of malignant cells.*
(X1100)

PLATE XVI

*Uniform enlargement of right optic nerve through infiltration by tumour.*

PLATE XVII

*Nerves in cauda equina showing focal expansions by tumour.*

PLATE XVIII

*Photomicrograph of section showing infiltration of cerebellum and meninges by poorly differentiated adenocarcinoma.*

(x60)

### A CASE OF MENINGEAL CARCINOMATOSIS

*A Clinical Pathological Conference of the University of Bristol Medical School*

(P.M. 6388)

CHAIRMAN: DR. O. C. LLOYD

(Though discussed at a Clinical Pathological Conference there was, unfortunately, no record of the proceedings kept. The case is therefore now presented as a report.)

*Dr. C. F. Allenby*, deputizing for Dr. J. E. Cates, presented the clinical picture: The patient, a man of 38, worked as a cable layer. In September, 1959, he was hit in the chest by a sledge-hammer and dated his illness from this injury. A week later he was admitted to Ham Green Hospital with pneumonia. Response to penicillin, and later to streptomycin, was slow and X-ray of his chest revealed a cavity in his right lung. He discharged himself against advice and did not attend for further X-rays. In October, 1959, when turning in bed he felt a sudden pain in his back which later spread down both legs. He was seen in the Orthopaedic Department and then referred to Dr. J. E. Cates. The only other feature in his history was that in 1957 he was off work for 8 months with pain in his back after an injury when lifting sacks at work.

In the Out-Patient Clinic he was seen to be thin and ill. There was clubbing of his fingers. There was 1 in. wasting of the right thigh with impairment of sensation over the right leg and depressed right knee jerk.

He was admitted for further investigation a few days later and developed in this short time fresh neurological signs and symptoms. His right eye had become blind; the left third cranial nerve was paralyzed. There was a slight left facial palsy; besides the lower motor neurone signs in the right leg there were now signs of right pyramidal damage. The patient had periods of mental confusion.

Radiological examination revealed a fibrotic lesion in the right upper zone of the lung with cavitation and shift to the right of the upper mediastium. Degenerative changes were noted in the fifth lumbar vertebra and sacrum, possibly osteophytes. Carotid angiograms were normal.

Sputum and gastric washings showed no acid-alcohol-fast bacilli. W.R. and Kahn negative. Lumbar puncture showed a clear fluid, not under pressure, with slow rise and fall on jugular vein compression.

*Dr. A. B. Weber* described the C.S.F. findings: The sugar was reduced to 25 mgm per cent, protein 76 mgm per cent with increased globulin, and a pleocytosis of 27 c/mm consisting of 63 per cent lymphocytes and 37 per cent "large mononuclears" which were considered to be malignant cells (Plate XV).

*Dr. Allenby* continued: Bronchoscopy was performed, but revealed no evidence of carcinoma. The patient died about 4 months after the onset of symptoms. During life several diagnoses were considered; the most likely seemed to be meningeal carcinomatosis, the primary source of which had not been localized.

*Dr. N. G. Sanerkin* then described the necropsy: The body was that of a well-developed rather wasted adult male, with early finger clubbing. The right pleural cavity was obliterated supero-posteriorly by dense fibrous adhesions, and there was a right basal serofibrinous pleurisy. A left tonsillar abscess was found, which proved to be streptococcal. The right lung showed an irregular area of collapse-consolidation in the posterior part of the upper lobe, with ill-defined areas of induration, bearing numerous necrotic cavities which did not contain pus. There was surrounding fibrosis and emphysematous bullae, with a large bullous sac (10 cm in diameter) at the apex. The right lower lobe showed well-developed bronchopneumonia with pleurisy.

The left lung was free from tumour, but showed generalized emphysema with apical fibrosis, patchy basal collopse, some oedema and bronchiolitis.

A few of the right hilar lymph nodes were enlarged and infiltrated by tumour. The tracheobronchial, paratracheal, mediastinal, axillary, para-aortic, paragastric, mesenteric and iliac lymph nodes were infiltrated by tumour. Extension of tumour from the paratracheal nodes had involved the right recurrent laryngeal nerve. A large lymph node below the carina showed early tumour infiltration but no fixation, much of the enlargement being due to reactive hyperplasia.

Small tumour deposits were found in both adrenals, both kidneys, left tonsil, spleen and rectum; but none were present in the liver or pancreas. Multiple tumour deposits were found in the bones, especially skull and vertebrae.

The most remarkable findings were in the meninges, brain, and cranial and spinal nerves. The dura was studded with small plaques of tumour, mostly in the falx cerebri and tentorium cerebelli. The brain showed numerous tumour deposits (the largest 2 cm in diameter), mainly cortical in distribution, but with a few nodules in and around the basal ganglia; they were mostly solid, greyish-white, with areas of central necrosis, but a few were clearly mucoid and translucent. Similar nodules were found in the cerebellum. The right optic nerve was considerably and uniformly enlarged by tumour (Plate XVI). Similar enlargement was noted in the left oculomotor nerve. Small tumour deposits were present in the cauda equina (Plate XVII), and higher up in and around the spinal nerves, but no deposits were found in the spinal cord itself. There was no intervertebral disc herniation.

Histological examination showed a rather poorly differentiated adenocarcinoma of the lung, growing in parts in loose collections of spheroidal cells, in others showing tubular and acinar differentiation, with occasional production of mucin, and a distinct tendency to alveolar spread. The cerebral metastases showed much more obvious mucoid change. Mengineal infiltration was patchy, and was especially noted around the cerebellum (Plate XVIII) and spinal cord. The right optic nerve, and the other enlarged nerves, were diffusely infiltrated by tumour. A section from the left retina also showed deposits of tumour.

*Necropsy diagnosis:* Mucoid adenocarcinoma of lung, with multiple metastases and meningeal carcinomatosis. Apical pulmonary fibrosis; bullous emphysema. Streptococcal tonsillar abscess. Terminal bronchopneumonia.

This case provided a fairly typical clinical example of "meningeal carcinomatosis" with typical C.S.F. findings, including a low glucose level and the presence of malignant cells; indeed the discovery of the latter was the first indication of a malignant process in the patient.

McMenemy and Cummings (1959) state that tumour cells are frequently found in the C.S.F. in meningeal carcinomatosis, especially when the cauda equina is involved. They stress that a low glucose level is highly suggestive of carcinomatous meningitis, and should stimulate search for tumour cells.

Jacobs and Richland (1951) and Fischer-Williams, Bosanquet and Daniel (1955) provide case reports and review the literature. Their criteria for the diagnosis of meningeal carcinomatosis are (i) the presence of metastatic carcinoma cells in the subarachnoid space and (ii) the absence of gross nodule formation in the central nervous system. They consequently exclude all those cases in which gross metastatic nodules are present in the central nervous sysyem. They emphasize that the condition forms a distinct clinical and pathological entity. Clinically, the prominent features include headache, varying degrees of dementia, constipation, cranial nerve palsies, especially blindness. The majority of cases are secondary to adenocarcinomas, usually of the gastro-intestinal tract or lung. Fischer-Williams *et al.* (1955) state that of fifty bronchial carcinomas with intracranial metastases only three were adenocarcinomas, and all three had produced diffuse leptomeningeal spread (one of them with cerebral deposits as well).

Heathfield and Williams (1956) record two cases, with fairly typical clinical histories; one of their cases, however, like the present case, had tumour nodules in the brain. From the clinical aspect, the present case was certainly an example of "meningeal carcinomatosis". Pathologically, too, it showed leptomeningeal dissemination, with infiltration of cranial and spinal nerves. Fischer-Williams *et al.* would exclude this case, since tumour nodules were present in the brain. Nevertheless, it is pertinent to suggest that the presence or absence of such nodules may be largely immaterial, the important factor being the behaviour of a given tumour in the leptomeninges and subarachnoid space, no matter whether it gets there exclusively or in conjunction with cerebral metastases.

### REFERENCES

Jacobs and Richland (1951). *Bull. Los Angeles Neurol. Soc.*, **16**, 335.
Fischer-Williams, Bosanquet and Daniel (1955). *Brain*, **78**, 42.
Heathfield and Williams (1956). B.M.J., **i**, 328.
McMenemy and Cummings (1959). *J. Clin. Path.*, **12**, 400.

## Figures and Tables

**Figure f1:**
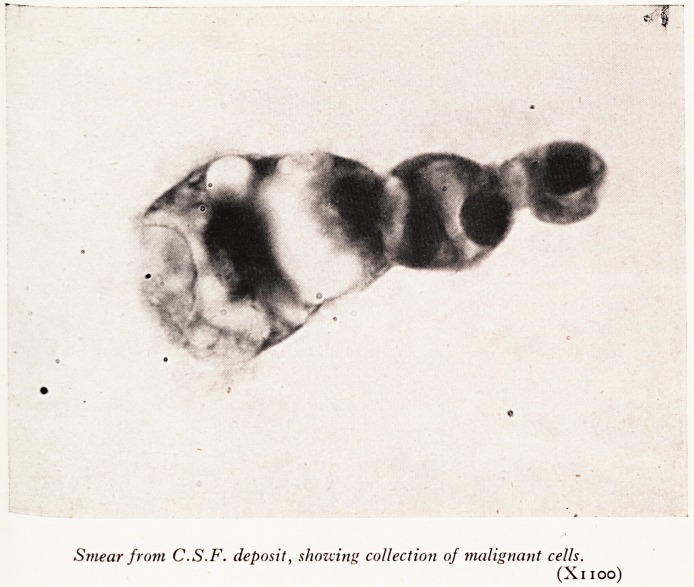


**Figure f2:**
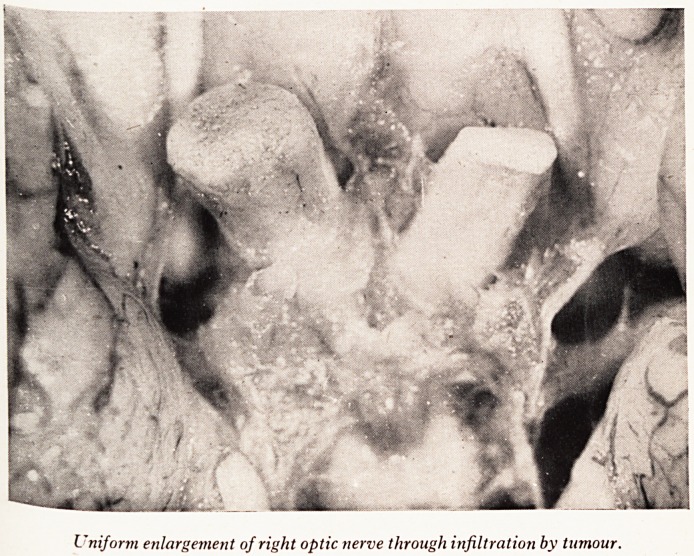


**Figure f3:**
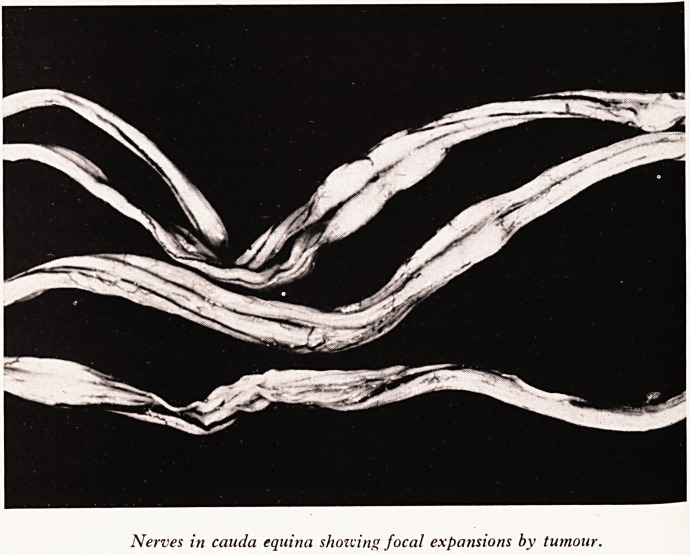


**Figure f4:**